# Early Prediction of Mortality after Birth Asphyxia with the nSOFA

**DOI:** 10.3390/jcm12134322

**Published:** 2023-06-27

**Authors:** Anne-Kathrin Dathe, Anja Stein, Nora Bruns, Elena-Diana Craciun, Laura Tuda, Johanna Bialas, Maire Brasseler, Ursula Felderhoff-Mueser, Britta M. Huening

**Affiliations:** 1 Neonatology, Paediatric Intensive Care and Paediatric Neurology, Department of Paediatrics I, University Hospital Essen, University of Duisburg-Essen, 45122 Essen, Germany; anne-kathrin.dathe@eah-jena.de (A.-K.D.);; 2 Centre for Translational Neuro- and Behavioural Sciences, C-TNBS, Faculty of Medicine, University of Duisburg-Essen, 45122 Essen, Germany; 3 Department of Health and Nursing, Occupational Therapy, Ernst-Abbe-University of Applied Sciences, 07745 Jena, Germany

**Keywords:** birth asphyxia, nSOFA, outcome prediction, neonate, hypoxic–ischemic encephalopathy (HIE), therapeutic hypothermia, resuscitation, organ dysfunction, critical illness assessment, mortality

## Abstract

(1) Birth asphyxia is a major cause of delivery room resuscitation. Subsequent organ failure and hypoxic–ischemic encephalopathy (HIE) account for 25% of all early postnatal deaths. The neonatal sequential organ failure assessment (nSOFA) considers platelet count and respiratory and cardiovascular dysfunction in neonates with sepsis. To evaluate whether nSOFA is also a useful predictor for in-hospital mortality in neonates (≥36 + 0 weeks of gestation (GA)) following asphyxia with HIE and therapeutic hypothermia (TH), (2) nSOFA was documented at ≤6 h of life. (3) A total of 65 infants fulfilled inclusion criteria for TH. All but one infant received cardiopulmonary resuscitation and/or respiratory support at birth. nSOFA was lower in survivors (median 0 [IQR 0–2]; *n* = 56, median GA 39 + 3, female *n* = 28 (50%)) than in non-survivors (median 10 [4–12], *p* < 0.001; *n* = 9, median GA 38 + 6, *n* = 4 (44.4%)). This was also observed for the respiratory (*p* < 0.001), cardiovascular (*p* < 0.001), and hematologic sub-scores (*p* = 0.003). The odds ratio for mortality was 1.6 [95% CI = 1.2–2.1] per one-point increase in nSOFA. The optimal cut-off value of nSOFA to predict mortality was 3.5 (sensitivity 100.0%, specificity 83.9%). (4) Since early accurate prognosis following asphyxia with HIE and TH is essential to guide decision making, nSOFA (≤6 h of life) offers the possibility of identifying infants at risk of mortality.

## 1. Introduction

The essential component to neonatal adaptation after birth is the initiation of adequate respiratory effort. Approximately 10–15% of newborns require support for respiratory transition at birth, 3% require positive pressure ventilation by mask, 2% intubation, and only <1% cardiopulmonary resuscitation with chest compressions or epinephrine to establish cardiorespiratory function [1,2]. The major cause for delivery room cardiopulmonary resuscitation is birth asphyxia, a condition of insufficient oxygen supply to vital organs that results in hypoxia, hypercarbia, and metabolic acidosis and, if prolonged, may progress to multiorgan failure, including the developing brain, which is then referred to as hypoxic–ischemic encephalopathy [3,4]. Asphyxia may originate from prenatal, perinatal or postnatal pathology. Prenatal maternal pathologies that increase the risk for birth asphyxia include diabetes mellitus or gestational diabetes, arterial hypertension, placental insufficiency, pregnancy toxemia, eclamptic seizure, infections, or drugs. Perinatal risk factors are, e.g., placental abruption, fetomaternal hemorrhage, amniotic fluid embolism, umbilical cord compression (knot or prolapse), insertio velamentosa of the umbilical cord, placenta previa, or shoulder dystocia. Postnatal causes of birth asphyxia are fetal anemia due to twin-to-twin transfusion in monochoriotic twins or fetal isoimmunization, airway anomalies, neurologic disorders, severe cardiopulmonary disease, infections, congenital malformations, intrauterine growth retardation, or medication effects.

Hypoxic–ischemic encephalopathy is a form of brain dysfunction (i.e., following brain injury) that occurs due to insufficient blood flow to the brain and/or insufficient oxygenation. The pattern of damage in hypoxic–ischemic encephalopathy depends on the severity, duration, and reoccurrence of hypoxia–ischemia and may result in involvement of the deep gray matter (basal ganglia and thalami), brainstem, and/or brain white matter in various combinations [5]. Hypoxic–ischemic encephalopathy is classified into three severity grades, according to Sarnat et al., based on clinical symptoms. For the diagnosis of moderate or severe hypoxic–ischemic encephalopathy (grade II and III), at least three of the six categories (e.g., vigilance, activity, reflexes, muscle tone, or apnea), i.e., a Sarnat Score ≥ 5, must be met [6]. While the Sarnat score is broadly used today, other scores, e.g., the Thompson score, with comparable clinical signs, exist.

Birth asphyxia accounts for 900,000 neonatal deaths worldwide annually and hypoxic–ischemic encephalopathy is estimated to cause up to a quarter of all postnatal deaths [7,8,9]. In developed countries, birth asphyxia occurs in 1.5–2.5 per 1000 live births and is one of the major causes for the development of cerebral palsy [10].

Therapeutic hypothermia is the only evidence-based neuroprotective therapeutic intervention currently available and is the standard of care in high income countries for moderate and severe hypoxic–ischemic encephalopathy (grade II and III) [11].

Therapeutic hypothermia improved the prognosis significantly: lower incidences of death and less severe cerebral palsy and epilepsy were reported in major randomized controlled trials on hypothermia [12,13,14,15]. However, mortality rates and the prevalence of severe disability are still high at 28% and 16–30%, respectively [11,16,17].

In severe hypoxic–ischemic encephalopathy, early prognosis is essential for parental counseling and treatment decisions, such as withdrawal of care. Currently available biomarkers and clinical parameters have in common that they require high personnel and technical resources. Furthermore, applicability and validity are limited in the first hours of life [18]. In a situation of a life-threatening illness, as severe hypoxic–ischemic encephalopathy, accurate and reliable determination of organ dysfunction and mortality risk is urgently needed.

The sepsis-related organ failure assessment (SOFA) score was designed to quantify organ dysfunction and mortality risk in adult intensive care patients with sepsis [19,20,21]. In recent years, its use is no longer limited to sepsis and the acronym is sometimes translated into sequential organ failure assessment, reflecting the broad dissemination of the SOFA score.

The neonatal modification of the SOFA (nSOFA) was proposed to address the need for a consensus definition of neonatal sepsis in 2020 [22]. nSOFA uses three objective and broadly available clinical parameters to quantify organ dysfunction: respiratory, cardiovascular, and hematological scores (total scores range from 0 to 15).

It was previously used for predicting mortality and severe morbidity in preterm infants [23], preterm infants with late onset sepsis [24], respiratory distress syndrome (RDS) [25], and neonates with proven sepsis [22,26]. Thus, the score was already shown to be predictive independent of the cause of organ dysfunction in the first 24 and 72 h.

The aim of the present study was to evaluate the accuracy of the nSOFA for predicting in-hospital mortality (sensitivity, specificity) following hypoxic–ischemic encephalopathy and therapeutic hypothermia.

## 2. Materials and Methods

### 2.1. Participants

For this retrospective study, the charts of all neonates with a gestational age of ≥36 + 0 weeks were reviewed who had received therapeutic hypothermia for hypoxic–ischemic encephalopathy following birth asphyxia at the level III NICU of the University Hospital Essen, Germany, between 1st December 2007 and 31st January 2023.

Therapeutic hypothermia was initiated based on clinical and laboratory criteria derived from large, randomized trials [12,13,14,15]. Therapeutic hypothermia after birth asphyxia was officially recommended by the American Heart Association in 2010, followed by the German Guidelines in 2013.

The eligibility criteria for therapeutic hypothermia at our institution did not change substantially in the evaluated time period and were as follows:

Birth asphyxia defined by at least one of the following criteria (block 1):

(a) documented severe acidosis with a pH ≤ 7.00;
(b) base excess of ≤ −16 mmol/l (blood from umbilical cord or neonate within the first 60 min of life);
(c) Apgar score ≤ 5 at 10 min of life;
(d) prolonged cardiorespiratory support (at least 10 min), consisting of chest compressions, epinephrine, intubation, bag and mask ventilation, or continuous positive airway pressure (CPAP).

In addition to moderate to severe hypoxic–ischemic encephalopathy defined by at least one of the following (block 2):

(a) encephalopathy (lethargy, stupor, coma) plus at least one of the following signs: (a1) muscular hypotonia, (a2) abnormal reflexes, or (a3) clinical seizures;
(b) post-2020 modified Sarnat score ≥ 5 (hypoxic–ischemic encephalopathy grade II and III);
(c) clinical seizures;
(d) pathologic amplitude integrates electroencephalogram (aEEG, discontinuous normal voltage, low voltage, burst-suppression, flat trace, or electroencephalographic seizures).

For inclusion in this retrospective study, a reconstruction of the Sarnat score was possible: five points for moderate encephalopathy was already present with invasive ventilation and a clinical neurological sign, such as muscular hypotonia.

Contraindications for therapeutic hypothermia were life-threatening congenital malformation (e.g., diaphragmatic hernia, cerebral malformation), suspected metabolic disease, coagulopathy with active bleeding, cerebral hemorrhage, cerebral venous thrombosis, hemorrhagic infarction, small for gestational age with birth weight < 1800 g, or severe pulmonary hypertension.

### 2.2. Therapeutic Hypothermia

Therapeutic hypothermia was delivered following a standardized protocol. Whole body hypothermia was initiated as early postnatally as possible but within 6 h after birth (TECOtherm^®^ tcmatt Neo, Tec Com Medizintechnik GmbH, Kabelsketal, Germany). A target temperature of 33.5 to 34.5 °C was monitored via a continuous rectal probe. It was sustained for 72 h followed by a rewarming phase. The temperature was increased 0.5 °C per hour until 37.0 °C was reached. Until 2015, this was performed manually; however, post-2015, a servo-controlled cooling mattress has been used.

### 2.3. Calculation of nSOFA

The nSOFA is calculated from 3 subcategories for respiratory, cardiovascular, and hematological status (Table 1). The respiratory category takes the status of mechanical ventilation, oxygen saturation (SpO_2_), and a fraction of inspired oxygen (FiO_2_) into account (score range 0–8). Cardiovascular status analyses the number of vasoactive drugs necessary to maintain normal blood pressure, including the use of corticosteroids (score range 0–4). The hematologic score is based on the presence and severity of thrombocytopenia (score range 0–3). The total score can therefore range from 0 (best) to 15 (worst) [22]. Data to calculate nSOFA in our cohort were derived from the patient charts, choosing the worst score value from the first 6 h of life, both values from the delivery room and after admission were included. Monitoring included continuous amplitude-integrated electroencephalogram (aEEG), a Thompson score (not standardized in time), and 1–2 hourly vital signs, including changes in ventilator settings. A complete blood count was included in the initial blood work on admission.

### 2.4. Endpoint: Death

The endpoint of the study was death. The timepoint of death and causes of death were taken from the medical records and/or death certificates. The decision to withdraw or limit care was based on a combination of clinical considerations—mainly the lack of respiratory effort in ventilated infants, severe CNS injury identified via Magnetic resonance imaging (MRI) or sonography, and severe organ dysfunction. If infants did not show respiratory effort after the cessation of mechanical ventilation, they were not reintubated and palliative care was initiated in the form of adequate analgesia, sedation, and/or weaning of cardiovascular support while maintaining enteral nutrition and warming for comfort.

### 2.5. Statistical Analysis

Analyses were performed using SPSS 29 (IBM Corp., New York, NY, USA). Patient demographics, clinical characteristics, and nSOFA scores are presented as medians and ranges or interquartile ranges for continuous variables and for categorical variables as counts and category percentages. Mann–Whitney U-tests and Fisher’s exact test were used to compare continuous and non-continuous data, respectively. Two-sided *p*-values < 0.05 were considered statistically significant. The Kaplan–Meier curve was used to visualize time to death and discharge.

Logistic regression was performed to calculate the odds ratio for in-hospital mortality with the nSOFA score as regressor. A receiver operating characteristics curve (ROC) was generated to plot true positive rate (sensitivity) against false positive rate (1—specificity) across varying threshold settings and areas under the receiver operating characteristic curves (AUCs) were calculated. An optimal cut-off value for odds of in-hospital mortality by nSOFA was determined using the Youden index and closest top-left methods. Positive and negative predictive values were calculated. 

## 3. Results

**Figure 1:**
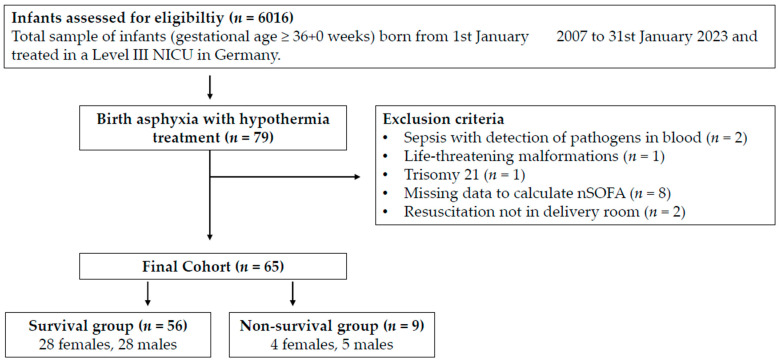
Inclusion and exclusion criteria. Notes: NICU = neonatal intensive care unit.

Out of the 6016 infants screened, 79 infants received therapeutic hypothermia due to hypoxic–ischemic encephalopathy (Figure 1). One infant, at 37 + 0 weeks of gestation, was included although the birth weight was 1745 g (contrary to recommendations of the American Heart Association) based on an individual treatment decision. We excluded four infants from the analysis because of a baseline condition with influences on organ function or mortality other than hypoxic–ischemic encephalopathy: two infants with blood culture-proven early onset sepsis, one infant with congenital malformation, and one infant with chromosomal abnormality. Two infants had life-threatening congenital malformations, so they were excluded. Two infants were excluded, since they did not suffer from birth asphyxia but required resuscitation later than in the delivery room. A total of 45 of 79 infants were inborn. Outborn infants were transferred to our institution and therapeutic hypothermia was begun within the first 6 h of life. Of these, Three infants were born outside of a hospital, which led to missing data in one case. Seven additional cases of outborn infants had to be excluded, because data to calculate nSOFA were not sufficient. This led to a final cohort of 65 infants with 21 outborn infants (survival cohort *n* = 16; non-survival cohort *n* = 5) (Figure 1).

All neonates fulfilled at least one of the criteria for birth asphyxia (block 1), 26.2% of infants presented with two criteria, and 64.6% with more than two criteria (Table 2).

All infants had at least moderate hypoxic–ischemic encephalopathy as indication for therapeutic hypothermia (block 2). A total of 47.7% infants presented with two criteria and 38.8% with more than two criteria. Clinical signs of hypoxic–ischemic encephalopathy according to criteria (a) were fulfilled by 60 infants; in 13 infants, the Sarnat score was available; and in 21 cases, the Sarnat score was reconstructed (invasive ventilation plus muscular hypotonia). Clinical seizures or pathological aEEG were valid for three and forty infants, respectively.

### 3.1. Clinical Characteristics

Survivors (*n* = 56) and non-survivors (*n* = 9) were similar with respect to gestational age, birth weight, small for gestational age, head circumference, child sex, umbilical cord pH, umbilical base excess (BE), and seizures before and during hypothermia treatment (Table 3). Maternal platelets in blood sample closest to birth showed no group differences (survivor group median 223 [119–385]/nL, non-survivor group median 197 [137–302]/nL). The platelets were less than 160/nL in three cases of each group. There was no platelet count of less than 100/nL. Neither pregnancy-associated nor immunological diseases were present in these mothers. In one case, an amniotic infection syndrome was present.

**Figure 2:**
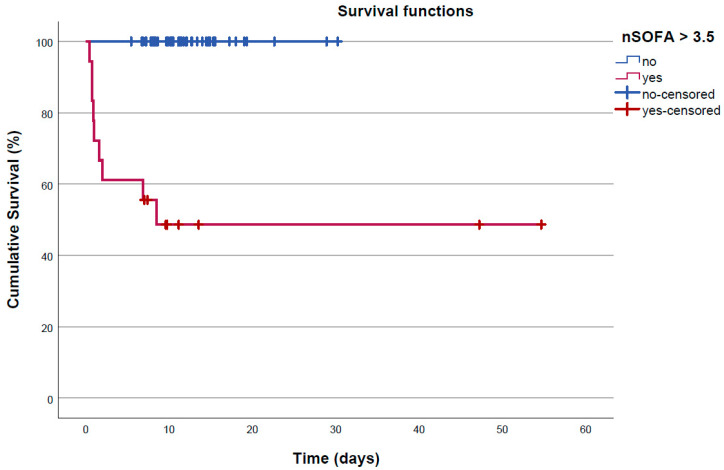
Kaplan–Meier curve of time to mortality or discharge (censor) using an nSOFA cut-off level (as reported later) of 3.5. Notes: The length of hospital stay did not differ between groups with an nSOFA value above 3.5 (*n* = 9, median 9.7 [range 7.0–54.7] days) and below 3.5 (*n* = 47, median 11.4 [5.4 to 30.2] days).

Non-survivors had lower pH and BE in the infant blood gas analysis during the first hour of life and lower Apgar scores at 1, 5, and 10 min. Non-survivors were more often born via c-section or emergency c-section (Table 3). A comparison of complete Sarnat scores was not possible due to the small number of cases. One surviving infant was born as a twin, but there were no multiple births in the non-survival cohort. All but one infant received cardiopulmonary resuscitation (chest compression and/or epinephrine) and/or respiratory support during transition after birth. Non-survivors were more likely to have received delivery room resuscitation and epinephrine during postnatal care. There was the need for respiratory support in both groups. However, there were more intubations in the non-surviving group while temporary mask ventilation via T-piece was sufficient for stabilization in the group of survivors (Table 3). Death occurred within the first 24 h in five neonates and before 48 h of life in another two neonates. Two infants died later on days 6 and 8 (Figure 2, Table 3). Causes of death were lack of respiratory effort in four cases, multi-organ dysfunction in four cases, and circulatory failure in one case. Four of the non-surviving infants received MRI. In survivors, the median length of hospital stay was 11.3 days with a wide range (Figure 2, Table 3).

### 3.2. nSOFA Scores in Survivors and Non-Survivors

**Figure 3:**
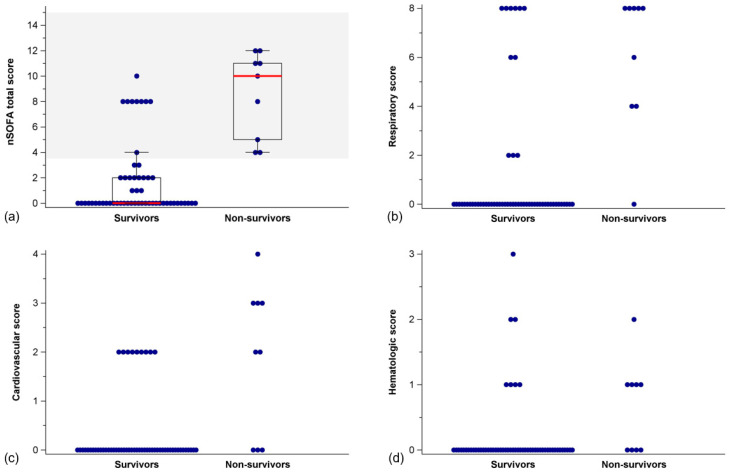
Distribution (scatter plot) of (**a**) nSOFA total scores with box plot (median, IQR), (**b**) respiratory scores, (**c**) cardiovascular scores, and (**d**) hematologic scores in survivors and non-survivors. Notes: The area above the nSOFA cut-off value of 3.5 is colored gray. nSOFA = neonatal sequential organ failure assessment; IQR = interquartile range.

nSOFA sum scores were lower in survivors than in non-survivors (Mann–Whitney U-test, *p* < 0.001) (Figure 3, Table 4). This also held true for respiratory (*p* < 0.001), cardiovascular (*p* < 0.001), and hematologic sub-scores (*p* = 0.003), respectively (Figure 3, Table 4).

### 3.3. Prediction of In-Hospital Mortality by nSOFA

**Figure 4:**
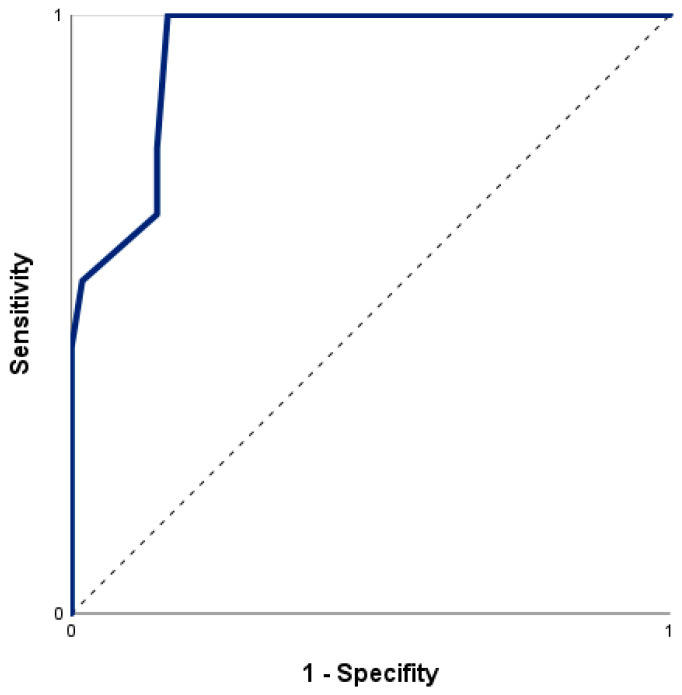
Receiver operating characteristics curve for nSOFA to predict non-survival.

A significant relationship of nSOFA and mortality was confirmed with an odds ratio for mortality of 1.61 [95% CI = 1.24–2.08] per one-point increase in nSOFA score (Χ^2^ (1) = 25.98, *p* < 0.001, Nagelkerkes R^2^ = 0.53). The ROC curve for risk of death by nSOFA (Figure 4) had an area under the curve (AUC) of 0.94 (95% CI = 0.88–1.00). The optimal cut-off value of the nSOFA score, according to the Youden index and the closest top-left method was 3.5 (sensitivity 100.0%, specificity 83.9%). Using a cut-off of 3.5 points on the nSOFA score, the positive and negative predictive values were 50.0% and 100.0%, respectively (cross-tabulation in Table 5).

## 4. Discussion

### 4.1. Prediction of In-Hospital Mortality

The nSOFA, as a critical illness assessment, proved useful for predicting in-hospital mortality in neonates with hypoxic–ischemic encephalopathy during therapeutic hypothermia. Non-survivors showed significantly higher sum scores, as well as respiratory, cardiovascular, and hematologic sub-scores. A one-point increase in nSOFA increased the odds for in-hospital mortality by 1.6. None of the infants with a nSOFA score < 3.5 died in this cohort (negative predictive value: 100%). Thus, the nSOFA serves well as an operational definition of organ dysfunction identifying neonates at risk for death following hypoxic–ischemic encephalopathy.

Despite the excellent negative predictive value of nSOFA scores of < 3.5, several survivors had nSOFA scores of 8, limiting the positive predictive value of the cut-off value. This is likely due to the fact that the respiratory sub-score has the highest weight in the nSOFA sum score, with mechanical ventilation contributing to 8 points of the possible total of 15. All non-survivors and 41% of survivors were intubated and received mechanical ventilation within the first 6 h of life, which resulted in respiratory sub-scores of 2 to 8 points. The notable effect of respiratory management on the nSOFA was also present in other studies within the first 72 h of life in very preterm infants, as well as in late onset sepsis regardless of gestational age [23,26].

### 4.2. Respiratory and Cardiovascular Sub-Scores

Factors contributing to the need for mechanical ventilation in hypoxic–ischemic encephalopathy and therapeutic hypothermia are manifold: Hypoxia at birth may prevent the onset of spontaneous breathing, lead to apnea and bradycardia, and prevent physiologic transition of circulation to extrauterine life, resulting in pulmonary hypertension and persistent fetal circulation [3]. Therapeutic hypothermia exerts direct effects on respiration by increasing pulmonary vascular resistance and reducing oxygen consumption and release (hemoglobin dissociation) [27]. Neuroactive medication, injury to the respiratory control center in the brain stem, and status epilepticus affect respiratory drive, potentially exacerbating hypoxia and disturbed CO_2_ elimination [28]. In a previous study, it was shown that the need for mechanical ventilation was significantly higher in the group with severe asphyxia and unfavorable outcomes (death and severe brain injury on MRI) compared to infants with better short-term outcomes [29]. However, in this cohort, the need for mechanical ventilation without another sign of organ dysfunction was not inextricably associated with death, calling for caution when interpreting nSOFA scores in neonates on mechanical ventilation who are otherwise stable.

Because the establishment of sufficient oxygenation and ventilation is the most important aspect of delivery room resuscitation, the fact that few neonates require chest compressions and/or the administration of epinephrine should not be misinterpreted. A neonate who experiences birth asphyxia may still develop multi-organ failure and become life-threateningly ill due to the redistribution of cardiac output to vital organs such as the brain, myocardium, and adrenal gland. Reduced perfusion to the other organs may cause local hypoxia/ischemia and may result in organ failure [30]. If birth asphyxia is prolonged, cardiovascular deterioration occurs that eventually causes myocardial dysfunction. The fact that the cardiovascular sub-score showed fewer differences between survivors and non-survivors in this study may indicate that this cohort was less affected, or that cardiovascular impairment in asphyxia is less common than in sepsis, for which the nSOFA was originally developed for.

### 4.3. Hematologic Sub-Score

Thombocytopenia may result from both asphyxia and therapeutic hypothermia. There is a reduced release of platelets from the bone marrow and an increased destruction of circulating platelets in birth asphyxia, and platelet dysfunction during therapeutic hypothermia. The nadir of platelet count is on the 3rd day of life following asphyxia and 5th day of life during therapeutic hypothermia, suggesting an additive effect of therapeutic hypothermia [31,32,33]. The influence of therapeutic hypothermia on the early nSOFA score is therefore unlikely. It is reasonable to assume that cardiovascular and hematological sub-scores of the nSOFA increase during the acute phase of post-resuscitation treatment before dropping again. Therefore, these sub-scores may well be important in the sequential use of the nSOFA. Maternal diseases may influence the fetal platelet count as well but the statistical power of this study was not sufficient to investigate these potential confounders.

### 4.4. Decision Making

However, the early determination of organ dysfunction and the risk of mortality is desirable. All infants in this study had hypoxic–ischemic encephalopathy and therapeutic hypothermia as an expression of moderate to severe brain injury. In such a serious situation, objective and easily accessible prognostic markers can help in parental counseling and decision making.

nSOFA scores > 3.5 were associated with early neonatal death. Five of the nine non-survivors in this study died within the first 24 h of life, and two further neonates died in the first 48 h. In this cohort, neonates who survived this initial critical period were no longer at increased risk thereafter, despite an initial nSOFA score of >3.5. All these early deaths were caused by lack of respiratory effort, circulatory failure, or multi-organ dysfunction. The fact that only four of the non-surviving infants in this study received an MRI (day 5–7) highlights that they presented with such severe symptomatology that the extent of cerebral damage was either not necessary for decision making to discontinue therapy or the infants died despite full therapy. This observation is in accordance with other reports on early neonatal deaths (<72 h), in the phase of clinical instability and critical illness [34,35].

Because this is the first application of the nSOFA score in HIE, it is too early to recommend clinical application at this point. Rather, these data generate the hypothesis that the nSOFA may be useful for classifying mortality risk in the decision-making process in infants with hypoxic–ischemic encephalopathy during therapeutic hypothermia if the results can be reproduced and validated in a larger, prospective multi-center study.

### 4.5. Available Biomarkers and Clinical Assessments

First-hour clinical parameters, such as umbilical cord pH and Apgar scores have limited reliability in predicting individual mortality. In addition, the Apgar includes subjective components with high inter-observer variability. Currently available biomarkers, physical examination, chemical, electrophysiological, and imaging studies all have specific limitations. Clinical examinations require experience and may be influenced by treatment/medication. Chemical biomarkers, e.g., plasma biomarkers, proteomics, and metabolomics, require specific lab resources and may have a delayed response to the injury [18]. Electrophysiology has a good predictive marker for abnormal brain activity, but it necessitates equipment, resources, and expertise [36]. MRI examinations also involves a large amount of time and effort, the risk of transporting a critically ill infant and limited accuracy of early scans compared to those obtained at the end of the first week of life [37]. Existing neonatal critical illness scores are either designed for very preterm infants (clinical risk index for babies—CRIB I and II; Berlin Score) are too complex and inconvenient for use and/or variables are collected over a longer period of time—up to 24 h after birth (score for neonatal acute physiology (perinatal extension)—SNAP I/II and SNAP-PE I/II; (extended) sick neonatal score—(E)SNS) [38,39]. Scores like Sarnat and Thompson may aid in decision making regarding hypothermia treatment or decision making. However, the results depend on the timing of scoring, as symptoms may evolve over time, and on the expertise of the examiner and may be less predictive of mortality, as our results show [40]. It is therefore desirable to have a very early and at the same time accurate assessment of prognosis, which can easily be performed without technical effort or special expertise.

### 4.6. Strengths and Limitations

The strength of our study lies in the fact that we were able to show for the first time that the nSOFA offers the potential to identify infants at risk of mortality following hypoxic–ischemic encephalopathy and therapeutic hypothermia within the first 6 h of life. The nSOFA is easy to apply, does not require a large number of human resources or anys technical equipment. It is based on variables that can be objectified and measured even in low-resource settings. The nSOFA may serve as a valuable tool in the process of decision making after severe birth asphyxia with hypoxic–ischemic encephalopathy and therapeutic hypothermia.

The nSOFA has already proven its suitability as a predictor for unfavorable outcomes in neonates diagnosed with sepsis. Therefore, neonates with sepsis, which must be considered a potential confounder, were excluded from our study. From clinical experience, an infant suffering from hypoxic–ischemic encephalopathy may also have sepsis. However, our study had a small sample size, which limits the statistical power to allow for subgroup analysis.

Other limitations of this study need to be recognized. This single-center study was performed retrospectively over a long period. This is due to the fact that the center, although providing the highest level of care, tends to care for a small annual number of infants with hypoxic–ischemic encephalopathy, compared to international standards, with many of them referrals. The German system of care is highly decentralized and special centers for asphyxia treatment do not exist.

Although inclusion criteria and major therapeutic regimes did not substantially change, it cannot be excluded that neonatal intensive care of infants has changed slightly over time.

## 5. Future Aspects and Conclusions

Therefore, the results of this study should be prospectively replicated in multiple centers and larger samples to investigate influencing factors such as maternal diseases, delivery mode, socio-economic status, and fetal factors, e.g., child sex, small for gestational age, and sepsis. In addition, it may be of interest to explore the extent to which the nSOFA is helpful in future decision making and in counseling with parents. The nSOFA is easy to apply, measurable even in low-resource settings, and might be used to identify infants at risk of in-hospital mortality due to hypoxic–ischemic encephalopathy and therapeutic hypothermia. Early accurate prognosis in hypoxic–ischemic encephalopathy during therapeutic hypothermia is essential for decision making.

## Figures and Tables

**Table 1:** Neonatal sequential organ failure assessment (nSOFA) [22].

Component	nSOFA Scores				
Respiratory score	0	2	4	6	8
	Not intubated or intubated SpO_2_/FiO_2_ ratio ≥ 300	Intubated SpO_2_/FiO_2_ ratio < 300	Intubated SpO_2_/FiO_2_ ratio < 200	Intubated SpO_2_/FiO_2_ ratio < 150	Intubated SpO_2_/FiO_2_ ratio < 100
Cardiovascular score	0	1	2	3	4
	No systemic corticosteroids and no inotropes	Systemic corticosteroid treatment but no inotropes	1 inotrope and no systemic corticosteroids	≥2 inotropes or 1 inotrope and systemic corticosteroids	≥2 inotropes and systemic corticosteroids
Hematologic score	0	1	2	3	
	Platelet count ≥150/nL	Platelet count 100–149/nL	Platelet count <100/nL	Platelet count <50/nL	

Notes: FiO_2_ = fraction of inspired oxygen; SpO_2_ = oxygen saturation as measured by pulse oximetry. Medications considered as inotropes (or vasoactive) = dopamine, dobutamine, epinephrine, norepinephrine, vasopressin, and milrinone.

**Table 2:** Eligibility criteria for therapeutic hypothermia (number (%)).

	Infants (*n* = 65)
Block 1 Birth asphyxia criteria	
(a) Severe acidosis, pH ≤ 7.00 ^a^	56 (91.8) ^c^
(b) Base excess ≤ −16 mmol/L ^a^	42 (68.9) ^c^
(c) Apgar 10 min ≤ 5	22 (34.4) ^d^
(d) Prolonged delivery room resuscitation ^b^	59 (96.7) ^c^
Number of criteria block 1:	
One	6
Two	17
>two	42
Block 2 Hypoxic–ischemic encephalopathy criteria	
(a) Encephalopathy (lethargy, stupor, coma) plus	60 (92.3)
(a1) Muscular hypotonia	51 (78.5)
(a2) Abnormal reflexes	58 (89.2)
(a3) Clinical seizures	3 (4.6)
(b) Sarnat score ≥ 5	34 (52.3)
(c) Clinical seizures	3 (4.6)
(d) Pathologic aEEG	40 (70.0) ^e^
Number of criteria block 2:	
One	15
Two	31
>two	20

Notes: ^a^ pH und base excess: Infant’s first blood sample was obtained arterial, venous, or capillary and derived either from cord blood or was drawn at admission to the NICU within the first hour of life. ^b^ Cardiopulmonary resuscitation of at least 10 min includes respiratory support, chest compressions, and Epinephrine. Descriptive statistics are based on ^c^ *n* = 61, ^d^ *n* = 64, and ^e^ *n* = 58, otherwise *n* = 65 infants.

**Table 3:** Clinical characteristics of survivors and non-survivors.

	Survivors (*n* = 56)	Non-Survivors (*n* = 9)
Gestational age, weeks	39 + 3 [36 + 0–41 + 4]	38 + 6 [37 + 2–41 + 2]
Weight at birth, grams	3170 [1745–4400]	3380 [2600–4400]
SGA, *n* (%)	12 (21.4)	1 (11.1)
Height at birth, cm	50.0 [42.0–58.0]	52.5 [50.0–56.0] ^f^
Head circumference at birth, cm	34.0 [30.0–38.0] ^g^	34.8 [31.5–38.0] ^f^
Female, *n (%)*	28 (50.0)	4 (44.4)
Multiple birth, *n* (%)	1 (1.8)	0 (0)
C-section, *n* (%)	28 (50)	8 (88.9)
Perinatal sentinel event ^a^, *n* (%)	22 (39.3)	7 (77.8)
Umbilical cord pH (SD)	6.98 [6.61–7.28] ^h^	7.05 [6.59–7.29] ^i^
First pH from infant ^b^	6.93 [6.75–7.19] ^j^	6.74 [6.41–7.23]
Umbilical cord base excess	−15.25 [−33.90–−4.20] ^k^	−16.70 [−23.00–−5.00] ^l^
First base excess from infant ^b^	−17.90 [−28.00–−9.10] ^m^	−25.15 [−31.70–−8.70] ^f^
Apgar 1 min	2 [0–6] ^g^	0 [0–1]
Apgar 5 min	5 [0–8] ^g^	0 [0–4]
Apgar 10 min	7 [1–10] ^g^	0 [0–4]
Delivery room resuscitation ^c^, *n* (%)	13 (23.2)	7 (77.7)
Respiratory support ^d^, *n* (%)	55 (98.2)	9 (100)
Intubation, *n* (%)	23 (41.0)	9 (100)
Mask ventilation via T-piece, *n* (%)	28 (50.0)	0 (0)
CPAP, *n* (%)	3 (5.4)	0 (0)
Chest compressions, *n* (%)	3 (5.3)	0 (0)
Epinephrine, *n* (%)	4 (7.3) ^g^	6 (66.7)
Sarnat score ^e^	11 [5–15] ^n^	20.5 [18–23] ^o^
Sarnat score ≥ 5 (reconstructed) ^e^, *n* (%)	25 (45.5) ^g^	9 (100)
Seizure activity in aEEG, *n* (%) Before TH, *n* (%)	2 (3.6) ^g^	0 (0)
During TH, *n* (%)	16 (29.1) ^g^	5 (62.5) ^f^
Time to death or discharge, days	11.29 [5.43–54.66]	0.97 [0.42–8.50]

Notes: Data are presented as median [range] if not indicated otherwise. SGA = small for gestational age with weight of birth < 10th percentile; cm = centimeter; CPAP = continuous positive airway pressure; TH = therapeutic hypothermia; C-section = caesarean section. ^a^ Perinatal sentinel events include: placental abruption, uterine rupture, umbilical cord trauma (either cord prolapse, knot, tear, rupture, or compression), shoulder dystocia, severe internal bleeding, trauma, cardiorespiratory arrest, or seizures immediately before birth, ^b^ pH und base excess: infant’s first blood sample was applied arterial, venous, or capillary and derived either from cord blood or was drawn at admission to the NICU within the first hour of life, ^c^ Delivery room resuscitation includes prolonged chest compression and/or respiratory support for at least 10 minutes postnatally. ^d^ Respiratory support applied in the delivery room, ^e^ Sarnat scores displayed here were obtained within the first 6 h of life. A Sarnat score of ≥ 5 was reconstructed if invasive ventilation and clinical neurological signs, such as muscular hypotonias were present. Descriptive statistics are based on ^f^ *n* = 8, ^g^ *n* = 55, ^h^ *n* = 52, ^i^ *n* = 7, ^j^ *n* = 47, ^k^ *n* = 44, ^l^ *n* = 4, ^m^ *n* = 45, ^n^ *n* = 11, and ^o^ *n* = 2, otherwise *n* = 56 in the survival group and *n* = 9 in the non-survival group.

**Table 4:** Descriptive characteristics of nSOFA total score and sub-scores in survivors and non-survivors.

	Survivors (*n* = 56)	Non-Survivors (*n* = 9)	*p*-Value
nSOFA total score	0 [0–2]	10 [4.5–11.5]	<0.001
Respiratory score	0 [0–0]	8 [4–8]	<0.001
Intubation, *n* (%)	23 (41.1%)	9 (100.0%)	<0.001
SpO_2_/FiO_2_ ratio	447.6 [246.9–461.9] ^a^	79.0 [55.0–178.3]	<0.001
Cardiovascular score	0 [0–0]	2 [2,3]	<0.001
One inotrope, *n* (%)	9 (16.1%)	2 (22.2%)	0.642
Two or more inotropes, *n* (%)	0 (0%)	4 (44.4%)	<0.001
Systemic steroids, *n* (%)	0 (0%)	1 (11.1%)	0.138
Hematologic score	0 [0–0]	1 [1]	0.003
Platelets/nL	237 [180–279]	132 [110–242]	0.077

Notes: Data are presented as median [interquartile range] if not indicated otherwise. Mann–Whitney U-test and Fisher’s exact test were used for continuous and non-continuous data, respectively. ^a^ *n* = 52.

**Table 5:** Cross-tabulation of nSOFA cut-off value of 3.5 in survivors (*n* = 56) and non-survivors (*n* = 9).

		Survivors	Non-Survivors	Total
nSOFA at least 3.5	no	47	0	47
	yes	9	9	18
		56	9	65

Notes: Data are presented as *n*.

## Data Availability

The dataset used and/or analyzed for the study is available from the corresponding author upon reasonable request.
